# Demand-resource evaluations and post-performance thoughts in classical music students: how they are linked and influenced by music performance anxiety, audience, and time

**DOI:** 10.3389/fpsyg.2025.1579759

**Published:** 2025-05-12

**Authors:** Ludovic Rey, Amélie J. A. A. Guyon, Horst Hildebrandt, Angelika Güsewell, Antje Horsch, Urs M. Nater, Jeremy P. Jamieson, Patrick Gomez

**Affiliations:** ^1^Department of Occupational and Environmental Health, Unisanté, Center for Primary Care and Public Health & University of Lausanne, Lausanne, Switzerland; ^2^Department of Clinical Research, Faculty of Medicine, University of Bern, Bern, Switzerland; ^3^Swiss University Center for Music Physiology, Basel University of the Arts, Basel, Switzerland; ^4^Swiss University Center for Music Physiology, Zurich University of the Arts, Zurich, Switzerland; ^5^HEMU–Haute Ecole de Musique, HES-SO University of Applied Sciences and Arts Western Switzerland, Lausanne, Switzerland; ^6^Institute of Higher Education and Research in Healthcare (IUFRS), University of Lausanne, Lausanne, Switzerland; ^7^Neonatology Service, Department Woman-Mother-Child, Lausanne University Hospital, Lausanne, Switzerland; ^8^Department of Clinical and Health Psychology, University of Vienna, Vienna, Austria; ^9^University Research Platform “Stress of Life (SOLE) – Processes and Mechanisms Underlying Everyday Life Stress”, University of Vienna, Vienna, Austria; ^10^Department of Psychology, University of Rochester, Rochester, NY, United States

**Keywords:** biopsychosocial model of challenge and threat, demand and resource evaluations, music performance anxiety, perseverative cognition hypothesis, post-event processing, postevent rumination, social-evaluative stress

## Abstract

**Introduction:**

Musicians’ performance experiences range widely, from elation to severe anxiety. In this study, we examined musicians’ performance experiences through the lens of the biopsychosocial model of challenge and threat. According to this model, a challenge state arises when perceived resources meet or exceed perceived demands, while a threat state occurs when demands outweigh resources. These states can be quantified using the Demand Resource Evaluation Score (DRES), calculated as the difference between resource and demand evaluations, with higher values indicating a greater challenge-type response. Although post-event processing is a key factor in maintaining social anxiety, research on factors influencing musicians’ post-performance thoughts remains limited. Additionally, the link between DRES and post-performance thoughts is unknown. This study aimed to determine (1) how DRES is influenced by the general music performance anxiety (MPA) level, audience presence, and time (pre-performance vs. during-performance); (2) how negative and positive post-performance thoughts are influenced by general MPA level and audience presence; and (3) whether DRES predicts post-performance thoughts.

**Methods:**

Classical music students (*N* = 121) with varying levels of MPA performed solo in a private and a public session. We assessed pre-performance and during-performance DRES, and negative and positive post-performance thoughts.

**Results:**

DRES decreased with increasing general MPA level, was lower in public than private sessions, and declined from pre-performance to during-performance. These effects were qualified by a three-way interaction: the effect of general MPA level was strongest before performing publicly, the audience effect was most pronounced at higher general MPA levels before performing, and the time effect was greatest at lower general MPA levels during public sessions. General MPA level was associated with more negative thoughts and fewer positive thoughts. Audience presence increased only negative thoughts. Higher during-performance DRES predicted fewer negative and more positive thoughts both intraindividually and interindividually, with pre-performance DRES showing similar interindividual effects.

**Discussion:**

These findings demonstrate the complex interplay of personal and situational factors in shaping musicians’ challenge and threat experiences. Moreover, high general MPA levels are associated with a general tendency toward more negative and fewer positive post-performance thoughts. Interventions fostering challenge-oriented appraisals may enhance musicians’ post-performance processing, potentially mitigating performance anxiety.

## 1 Introduction

“Performers of all sorts, whether musicians, entertainers, actors, or public speakers love the liberating effects of challenge and hate the constricting effects of threat” (Lazarus, 1999, p. 76).

Performance lies at the heart of a musician’s career and is central to the aspirations of both professionals and music students. Achieving excellence in this complex endeavor demands advanced skills (Altenmüller and Ioannou, 2016). Additionally, musicians must navigate the pressures of public performance, often subject to evaluation. Therefore, performing can be psychophysiologically demanding, frequently triggering intense emotional, cognitive, behavioral, and physiological responses (Steptoe, 2001; Kenny, 2011; Studer et al., 2012; Sokoli et al., 2022). The pursuit of excellence under such conditions exposes musicians to stress, with some experiencing significant levels of music performance anxiety (MPA). MPA, defined as “the experience of marked and persistent anxious apprehension related to musical performance” (Kenny, 2010, p. 433), is a widespread phenomenon among classical music students and professionals (Studer et al., 2011; Fernholz et al., 2019). While it can occur in various contexts, it tends to be more pronounced in high-stakes situations characterized by ego involvement, evaluative pressure (e.g., audience presence), and a heightened fear of failure (Kenny, 2010; Fancourt et al., 2015; Aufegger and Wasley, 2018; Guyon et al., 2020a). MPA has recently been conceptualized as a functional response to adversity, with adversity being viewed as the combination of personality traits linked to advanced musical training, navigating the demands of high-pressure performances, and competitive and insecure professional settings (Herman and Clark, 2023).

We have suggested that the biopsychosocial model of challenge and threat could offer a valuable theoretical framework to investigate the psychophysiology of music performance and MPA (Guyon et al., 2020b). This model has been adopted as a theoretical framework in various high-pressure environments such as elite sport, aviation settings, and healthcare (Turner et al., 2013; Vine et al., 2015; Peek et al., 2023). Grounded in the transactional model of stress and coping (Lazarus and Folkman, 1984; Lazarus, 1991) and the concept of physiological toughness (Dienstbier, 1989), this model provides a framework for understanding the processes underlying motivated performance situations, which encompass contexts like test-taking, athletic competitions, and music performances. Such situations require individuals to produce instrumental responses to achieve self-relevant goals. The model posits that given task engagement, individuals may experience either challenge or threat depending on their evaluation of situational demands relative to their personal resources. A challenge state emerges when perceived resources meet or exceed the perceived demands of the situation, while a threat state arises when demands are perceived to outweigh available resources These states exist on a continuum, rather than as distinct binary opposites, influenced by both deliberate and automatic evaluations (Blascovich and Mendes, 2000; Weisbuch-Remington et al., 2005; for more background on the model see Blascovich, 2008; Seery et al., 2009; Seery, 2013; Jamieson, 2017). Factors that may enter into the demand-resource evaluation calculus include but are not limited to psychological and physical safety, uncertainty, novelty, skills, knowledge, required effort, presence of others, affective cues, attitudes, and beliefs (Blascovich, 2008; Moore et al., 2014).

At an experiential level, challenge and threat states can be captured using the Demand Resource Evaluation Score (DRES), a widely used measure defined as the difference between resource evaluation and demand evaluation (Moore et al., 2014; Wojcik et al., 2021; Peek et al., 2023). DRES is also known as resources-demands differential (Guyon et al., 2020b; Bosshard et al., 2023).

Demand and resource evaluations have been studied in the context of social anxiety and social threat. Gramer et al. (2012) found that a videorecorded speech task induced more threat-like evaluations in high socially anxious participants than in low socially anxious participants. Jamieson et al. (2013) used a between-subjects design in which socially anxious individuals and controls delivered a videotaped speech either to two interviewers providing negative nonverbal feedback throughout or in a private setting. Both groups perceived public speaking as more demanding than private speaking. Moreover, anxious participants experienced both tasks as more demanding than non-anxious participants, with the difference being larger in the evaluative condition. Anxious participants also perceived themselves as less resourceful than their non-anxious counterparts, and participants in the evaluative condition reported fewer resources than participants in the private condition.

In the domain of music performance, demand and resource evaluations as framed by the biopsychosocial model of challenge and threat remain unexplored. Nevertheless, Craske and Craig (1984) examined a related construct, measuring anxious and non-anxious pianists’ expectations of successfully completing performance-related tasks prior to performing privately and publicly. Anxious pianists reported lower expectations than non-anxious pianists. Additionally, among anxious pianists, expectations were lower in public performance compared to private performance, whereas no such difference was observed among non-anxious pianists. However, interpreting these results is complicated by the study design, as all participants performed privately before performing publicly, potentially confounding the effects of performance context. More recently, Osborne and McPherson (2019) showed that higher pre-recital self-perceived coping potential, assessed using an adapted version of the Precompetitive Appraisal Measure (Wolf et al., 2015), predicted less somatic and cognitive anxiety, more facilitative interpretations of somatic anxiety, and greater self-confidence assessed at the same time. Although their analyses did not examine predictors such as audience presence or general MPA level, their findings underscore the critical role of cognitive appraisals in shaping psychological responses to performance. These insights provide a foundation for the present study, which seeks to extend this line of inquiry by investigating demand and resource evaluations within the framework of the biopsychosocial model of challenge and threat.

Although challenge and threat states are considered dynamic (Blascovich, 2013), most studies have focused solely on anticipatory demand-resource evaluations (e.g., Moore et al., 2014; Vine et al., 2015). However, a few exceptions highlight their evolving nature. Gramer et al. (2012) reported a significant shift toward greater threat from before to during a videotaped speech task among high socially anxious participants, a trend not observed among those with low social anxiety. Aldao et al. (2014), Yeager et al. (2016), and Jacquart et al. (2020) reported an increase in threat from before to during the Trier Social Stress Test (Kirschbaum et al., 1993). Collectively, these findings indicate that DRES decreases from pre-performance to during-performance.

Perseverative cognition is defined as the “repetitive or sustained activation of cognitive representations of past stressful events or feared events in the future” (Brosschot et al., 2010, p. 407). According to the perseverative cognition hypothesis, perseverative cognition affects key stress systems and can contribute to poor health outcomes, including cardiovascular problems, mood disturbances, and psychosomatic complaints (Kubzansky et al., 1997; Holman et al., 2008; Jellesma et al., 2009; Verkuil et al., 2012; Ottaviani et al., 2016). In its original form, the perseverative cognition hypothesis remains silent regarding the role of the valence of the stressor-related cognitive representations (Smyth et al., 2013). Valence of the thought content is a critical determinant of cognitive processes (Watkins, 2008), with both negative and positive perseverative cognition playing significant roles in response to psychosocial stressors (Abbott and Rapee, 2004; Kocovski et al., 2011; Gramer et al., 2012; Donohue et al., 2021). An extended perseverative cognition hypothesis, which differentiates between negatively and positively valenced perseverative cognition, offers a promising avenue to better understand stress-related psychophysiological phenomena. This perspective may also enhance our knowledge of the effects of repeated exposure training under pressure, which has been shown to influence stress adaptation and resilience (Candia et al., 2023; de Bie et al., 2024).

In the social anxiety literature, the process of mentally reviewing a performance or social situation is referred to as post-event rumination or post-event processing (Watkins, 2008). It features prominently in many cognitive models of social anxiety disorder (see Flynn and Yoon, 2025, for review). Most definitions of post-event processing consider it inherently negative and do not distinguish between positive and negative post-event processing (Flynn and Yoon, 2025). Research consistently shows that socially anxious individuals report more negative thoughts following a speech or conversation compared to non-anxious individuals (see Edgar et al., 2024 for review and meta-analysis). This perseverative, negative, self-referential thinking after social situations contributes to the maintenance of social anxiety (Clark and Wells, 1995; Brozovich and Heimberg, 2008; Rowa et al., 2016; Gavric et al., 2017; Katz et al., 2019). In contrast, few studies have investigated positive post-event thoughts, with some studies finding no significant effects of social anxiety (Edwards et al., 2003; Abbott and Rapee, 2004; Dannahy and Stopa, 2007) and others reporting significantly fewer positive thoughts among socially anxious individuals than non-anxious individuals (Kocovski et al., 2011; Gramer et al., 2012; Kane et al., 2023).

In the context of music performance, Nielsen et al. (2018) found that following a public solo performance, students with high general MPA level reported more negative thoughts (e.g., “I made a lot of mistakes”) and fewer positive thoughts (e.g., “My concert was good”) than students with low general MPA level. Highlighting the significance of both negative and positive post-performance thoughts for musicians’ health and wellbeing, Haccoun et al. (2020) demonstrated that negative post-performance thoughts predicted higher daily cortisol output, whereas positive post-performance thoughts predicted lower daily cortisol output. Cortisol is a key stress hormone, making it particularly relevant in understanding the biological impact of post-performance thought patterns. However, these findings are limited to public performance settings, leaving it unclear whether these effects extend to private performance situations. The present study addresses this gap by investigating how general MPA level and audience presence influence both negative and positive post-performance thoughts.

Finally, the present study proposes an integrated framework linking the biopsychosocial model of challenge and threat with the extended perseverative cognition hypothesis in the context of music performance. This novel framework posits that higher (vs. lower) DRES predicts fewer (vs. more) negative post-performance thoughts and more (vs. fewer) positive post-performance thoughts. Supporting this idea, Gramer et al. (2012) found that participants’ pre-task perceived demand-to-resource ratio (i.e., reverse scored DRES) significantly correlated with post-speech negative and positive thoughts. By integrating these perspectives, the present study seeks to advance our understanding of music performance and stress research through a bridge-building scientific approach. A glossary of key concepts is provided in Supplementary Table S1.

This study had three objectives. The first aim was to investigate to what extent DRES varies as a function of three factors: participants’ general MPA level, the performance context (private performance session vs. public performance session), and time (before the performance vs. during the performance). We hypothesized that DRES would be lower in the public performance session than the private performance session (main effect of session). In addition, we hypothesized that higher general MPA levels would be associated with lower DRES, particularly in the public performance session (general MPA level x session interaction). Furthermore, we hypothesized that DRES would be lower during performances than before (main effect of time). Whether the effects of general MPA level and session would depend on time was treated as an exploratory issue.

The second aim was to examine to what extent negative and positive post-performance thoughts are influenced by participants’ general MPA level and the audience context. We hypothesized that participants would report more negative thoughts and fewer positive thoughts following the public performance than the private performance (main effect of session). Moreover, we anticipated that higher general MPA levels would be associated with more negative thoughts and fewer positive thoughts, particularly following the public performance (general MPA level x session interaction).

Finally, the third aim was to determine whether pre-performance and during-performance DRES predict negative and positive post-performance thoughts at the within-person and between-person levels. We expected that higher DRES would predict fewer negative thoughts and more positive thoughts at both levels of analysis, thus supporting the proposed integrated framework linking the biopsychosocial model of challenge and threat with the extended perseverative cognition hypothesis.

## 2 Materials and methods

The data for this study were gathered as part of a psychophysiological study on music performance. For further information, see the study protocol article (Guyon et al., 2020b).

### 2.1 Participants

The study sample comprised 121 students enrolled in classical music programs at Swiss university music schools. The sample included 34 woodwind players, 31 string players, 23 singers, 14 pianists, 13 brass players, five guitarists, and one accordionist. Descriptive statistics of the sample relevant to the present study are reported in Table 1.

**Table 1:** Descriptive statistics of the sample.

	*N*	*M*	*SD*	Min–Max
Sample size	121			
Gender				
Males	52			
Females	69			
MPA		47.7	11.1	[27, 76]
Depressive symptoms		10.0	7.8	[0, 33]
Age (years)		24.3	3.2	[18, 33]
Time difference (days)		67.3	63.8	[6, 425]
Preparation (hours)		1.4	1.3	[0, 7]
Performance session order				
Private–Public	57			
Public–Private	64			
Time of day				
Early afternoon (1:00 p.m.)	62			
Late afternoon (3:45 p.m.)	59			

MPA = general music performance anxiety level; Time difference = days between the habituation session and the first performance session; Preparation = hours spent to practice the piece between the first and the second performance.

Eligibility was assessed through an online questionnaire. Participants who completed all phases of the study protocol received a remuneration of 250 Swiss francs and reimbursement for travel expenses. The study protocol was approved by the ethics committee of the canton of Vaud, Switzerland (protocol number 2019–01222).

### 2.2 Procedure

Participants were recruited via social media and the website of the HEMU-Haute Ecole de Musique in Lausanne, Switzerland. Interested students contacted the research team and were given a link to an online survey, which collected sociodemographic, academic, musical, and health-related data, as well as the general MPA level.

Of the 217 students initially expressing interest, 34 did not proceed beyond the first questionnaire. Participants were excluded based on the following criteria (number of excluded individuals in parentheses): age, which had to be between 18 and 35 years (2), enrollment in non-classical music programs (7), playing non-orchestral instruments, the harp, or the percussions (5), recreational drug use or medication, except hormonal contraception (3), and conditions affecting the cardiovascular, nervous, or endocrine systems (5). Additional exclusions included high scores for panic disorder (8) or eating disorders (7) on the Patient Health Questionnaire (for English, Spitzer et al., 1999; for French, Carballeira et al., 2007). Pregnancy, lactation, night-shift work, and pacemaker use were also exclusion criteria (1 for pacemaker use). Finally, no appointments could be scheduled with 16 participants, and eight participants only completed the habituation session.

Participants completed three laboratory sessions: a habituation session and two solo performance sessions. The habituation served two primary purposes: to familiarize participants with the experimental setup and to allow them to choose an instrument-specific piece from standard exam and audition repertoires to perform during the performance sessions (see Guyon et al., 2022, for the complete list of selected pieces).

The performance sessions were conducted 2 days apart, at the same time of day – either early afternoon (arrival at the lab at 1:00 p.m., performance at 2:00 p.m.) or late afternoon (arrival at 3:45 p.m., performance at 4:45 p.m.). The order of sessions was counterbalanced across participants. In both sessions, participants performed the same piece from memory without accompaniment. In the private session, they performed alone; in the public session, they performed before an audience of six to eight individuals, including the experimenter and two expert raters. Performance durations ranged from 2 min 36 s to 8 min 31 s (*M* = 4 min 10 s, *SD* = 45 s).

Prior to each session, participants were instructed to avoid alcohol and intense physical activity (24 h prior), heavy meals and caffeine (1 h 15 min prior), smoking (1 h prior), and food intake (15 min prior). A questionnaire assessing depressive symptoms was completed online 1 week after the second performance. The study was conducted in French for 108 participants and in English for 13 participants.

### 2.3 Questionnaires

Questionnaires were administered using the EFS Survey software (© UNIPARK & QuestBack, Germany). Sociodemographic, health, and academic information were assessed as described in Guyon et al. (2020b).

#### 2.3.1 General MPA level

Following previous work (Widmer et al., 1997; Kokotsaki and Davidson, 2003; Kim, 2005; Studer et al., 2012; Nielsen et al., 2018), students’ general MPA level was measured using the state scale of the State–Trait Anxiety Inventory (STAI-S; for English, Spielberger, 1983; for French, Spielberger et al., 1993). This scale contains 20 items such as “I am tense,” rated on a 4-point Likert scale (1 “not at all” to 4 “very much so”). Total scores range from 20 (no anxiety) to 80 (severe anxiety). Consistent with the performance situation of our study, participants were instructed to refer on how they generally feel when performing solo. Cronbach’s alpha and McDonald’s omega were as follows: English, *α* = 0.92, *ω* = 0.93; French, α = 0.93, ω = 0.93.

#### 2.3.2 Demand and resource evaluations

Demand and resource evaluations were measured with a widely used two-item instrument adapted for the music performance context (Moore et al., 2014; Peek et al., 2023). Pre-performance demand evaluation and resource evaluation were collected a few minutes before the performance using the questions, “How demanding do you expect this music performance situation to be?” and “How able are you to cope with the demands of the music performance situation?,” respectively. During-performance demand evaluation and resource evaluation were assessed a few minutes after the performance with the questions, “How demanding was the music performance situation?” and “How able were you to cope with the demands of the music performance situation?,” respectively. Participants answered on a 6-point Likert scale, ranging from 1 (“not at all”) to 6 (“extremely”). As is standard in the literature, the DRES was calculated by subtracting the demand score from the resource score for both pre- and post-performance assessments. DRES values range from −5 to +5, with higher values indicating a greater challenge-type response (Moore et al., 2014).

#### 2.3.3 Negative and positive post-performance thoughts

Negative and positive post-performance thoughts were assessed approximately 45 min after the end of each performance using the Post-Music Performance Thoughts Questionnaire (Nielsen et al., 2018). We assessed negative thoughts with 12 items (e.g., ‘I made a lot of mistakes’) and positive thoughts with 9 items (e.g., ‘My concert was good’). We excluded two items from the original 14-item negative thoughts subscale because they reference the audience, making them unsuitable for the private session. Participants rated the extent to which they had experienced each thought since the end of the performance on a 5-point Likert scale, ranging from 1 (“not at all”) to 5 (“very much so”). Separate mean scores, ranging from 1 to 5, were calculated for negative and positive thoughts. Higher scores represent more thoughts. Cronbach’s alpha and McDonald’s omega for negative thoughts were as follows: private session English, *α* = 0.91, *ω* = 0.92; private session French, *α* = 0.91, ω = 0.91; public session English, *α* = 0.85, ω = 0.85; public session French, *α* = 0.87, ω = 0.87. For positive thoughts, the reliability scores were as follows: private session English, *α* = 0.97, ω = 0.97; private session French, *α* = 0.93, ω = 0.93; public session English, *α* = 0.97, ω = 0.97; public session French, *α* = 0.94, ω = 0.94.

#### 2.3.4 Depressive symptoms

Depressive symptoms, a potential control variable, were measured using the Beck Depression Inventory-II (for English, Beck et al., 1996; for French, Beck et al., 1998). This 21-item questionnaire evaluates depressive symptoms over the past 2 weeks. Each item offers four statements, scored from 0 (least indicative of depression, e.g., “I do not feel sad”) to 3 (most indicative of depression, e.g., “I am so sad or unhappy that I cannot stand it”). Total scores range from 0 to 63, with higher scores reflecting more severe depressive symptoms. Reliability indices were as follows: English, *α* = 0.85, ω = 0.83; French, *α* = 0.89, ω = 0.89.

#### 2.3.5 Preparation time

Preparation time (in hours), a potential control variable, was measured at the end of each performance session with the following question “How much time have you spent in the last 48 h specifically preparing the musical piece you have just performed?”

### 2.4 Statistical analysis

Data were complete for all participants.

#### 2.4.1 Predictors of DRES and post-performance thoughts

To address the first two aims of the study, we conducted two-level mixed-effects linear regressions using STATA version 18.0 for Windows (Stata Statistical Software; StataCorp LP, College Station, TX). For the dependent variable DRES, the predictors of interest were general MPA level, session (private vs. public), and time (before vs. during). Specifically, we considered the main effects of these three variables, their three two-way interactions general MPA level x session, general MPA level x time, session x time, and their three-way interaction. For negative thoughts and positive thoughts, the predictors of interest were general MPA level and session, with their main effects and interaction. We also analyzed demand evaluation and resource evaluation separately. The results of these secondary measures are reported in the Supplementary Tables S2, S6–S9 and are not discussed here to maintain focus on the three primary outcomes.

Additionally, the following person- and design-related variables were examined as potential control variables: gender (females vs. males), age, depressive symptoms, time difference (days between the habituation session and the first performance session), preparation (hours spent to practice the piece between the first and the second performance), performance session order (private-public vs. public-private), and time of day (early afternoon vs. late afternoon). These variables were tested for their predictive value individually as a main effect and, except for preparation, in interaction with session and time. Effects with *p*-values below 0.05 were retained for the main analyses. The effects of these variables are not discussed to maintain focus on the effects of interest. All categorical variables were effect coded.

The random effect structure of the models was optimized using likelihood-ratio tests, Akaike information criterion (Akaike, 1973), and Bayesian information criterion (Schwarz, 1978). All models included a random intercept for participants. The residual variance structure was heterogeneous for DRES (distinct variance for each session and time) and homogeneous (i.e., one common variance) for negative and positive thoughts. Model assumptions were checked visually, using QQ-plots for residuals and random effect plots, and were found to be satisfactorily met.

Final models were run using restricted maximum likelihood estimation and the Kenward-Kroger approximation method for computing degrees of freedom in the *t* distribution.

#### 2.4.2 Links between DRES and post-performance thoughts

The links between DRES and negative and positive post-performance thoughts were analyzed using two-level path analyses in M*plus* for Windows version 8.11 (Muthén and Muthén, 1998-2017). A first analysis tested pre-performance DRES as a predictor of both negative and positive thoughts, while a second analysis tested during-performance DRES as a predictor. We specified direct paths from DRES to both types of thoughts at the within-person and between-person levels. The models were estimated using Bayes estimation, employing Markov chain Monte Carlo (MCMC) algorithms, which separate the within-person and between-person effects using latent decompositions. Two independent MCMC chains were used. Models with increasing complexity including random coefficient (slope) effects, random residual variances, and allowing for residual correlations between effects were tested, and the model with the lowest Deviance Information Criterion was selected as the final best-fitting model. A thinning factor of 50 was applied to reduce the autocorrelation among subsequent MCMC draws. The results are based on the posterior distribution of 20,000 iterations.

Convergence was assessed using the Potential Scale Reduction (PSR) criterion, where values close to 1 indicate good convergence (Gelman et al., 2004). Additionally, we examined posterior parameter trace plots and autocorrelation plots to evaluate the chain stability and mixing process, respectively.

We report both unstandardized and standardized point estimates, representing the median of the posterior parameter distribution, along with their associated 95% highest posterior density credibility intervals (HPD-CIs; Gelman et al., 2004). Parameters were considered statistically significant if their 95% HPD-CIs did not contain zero. Standardized coefficients indicate the change in the outcome variable associated with a one *SD* change in the predictor. For interpretation, we consider values below 0.30 as small effects, between 0.30 and 0.49 as medium effects, and above 0.50 as large effects (Cohen, 1988).

## 3 Results

### 3.1 Demand resource evaluation score (DRES)

Descriptive statistics for DRES are reported in Table 2. Preliminary analyses of potential control variables revealed a significant main effect of gender (see Supplementary Table S3), which was thus added to the main model alongside general MPA level, session, time, and their interactions.

**Table 2:** Descriptive statistics of the dependent variables across private and public sessions.

	Session	Time point	*M*	*SD*	Min–Max	95% CI
DRES	Private	Before	1.28	1.65	[−2, 5]	[0.98, 1.58]
		During	0.74	2.01	[−5, 5]	[0.37, 1.10]
	Public	Before	0.55	1.58	[−3, 5]	[0.27, 0.84]
		During	0.11	1.61	[−3, 5]	[−0.18, 0.40]
Negative thoughts	Private	After	1.65	0.65	[1.00, 3.92]	[1.53, 1.77]
	Public	After	1.83	0.62	[1.00, 3.75]	[1.72, 1.94]
Positive thoughts	Private	After	2.95	0.94	[1.11, 5.00]	[2.78, 3.12]
	Public	After	2.97	0.93	[1.00, 5.00]	[2.80, 3.13]

DRES = Demand Resource Evaluation Score; Before, During, and After refer to three time points relative to the performances.

**Figure 1:**
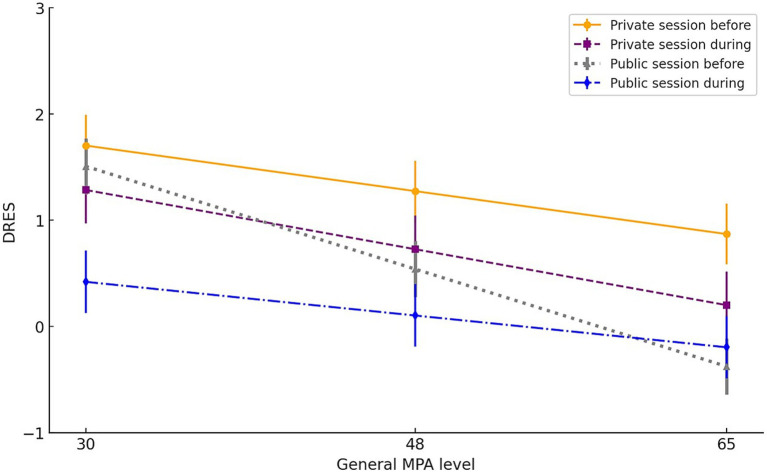
Model-estimated DRES means from low to high general MPA level for the four combinations of session (private vs. public) and time (before vs. during). Bars represent *SE*s. The values 30, 48, and 65 correspond to the 5th percentile, the mean, and the 95th percentile, respectively. These numbers are provided for illustrative purposes only, to help the reader interpret the range of general MPA levels in the sample. The slope coefficients are reported in Table 4.

As shown in Table 3, the main effects of general MPA level, session, and time were all significant. DRES decreased with increasing general MPA level, was lower in public than private sessions, and declined from pre-performance to during-performance. Importantly, these effects were further qualified by a significant three-way interaction. The model-estimated DRES means for the four combinations of session and time, plotted across levels of general MPA, are illustrated in Figure 1.

**Table 3:** Fixed effects of the final model for DRES.

	Coefficient	*SE*	*t*	*p*
MPA	**−0.032**	**0.012**	**−2.74**	**0.007**
Session	**−0.678**	**0.102**	**−6.65**	**< 0.001**
Time	**−0.496**	**0.102**	**−4.87**	**< 0.001**
Gender	0.283	0.255	1.11	0.27
MPA × session	−0.008	0.009	−0.90	0.37
MPA × time	0.015	0.009	1.57	0.12
Session × time	0.099	0.204	0.49	0.63
MPA × session × time	**0.043**	**0.018**	**2.35**	**0.019**

MPA = general music performance anxiety level. For Session, the reference is the private session. For Time, the reference is before the performance. Significant effects of interest are highlighted in bold.

To interpret the significant three-way interaction, we performed post-hoc analyses examining the significance of the three two-way interactions and the three main effects across different conditions.

Two-way interactions: We found that the general MPA level x session interaction was significant before the performance (coefficient = −0.030, *SE* = 0.012, *p* = 0.009) but was not significant during the performance (coefficient = 0.013, *SE* = 0.015, *p* = 0.35). The general MPA level x time interaction was significant during the public session (coefficient = 0.036, *SE* = 0.012, *p* = 0.002) but was not significant during the private session (coefficient = −0.007, *SE* = 0.014, *p* = 0.61). Finally, the session x time interaction was significant for general MPA levels below 23 and above 57 but was not significant for general MPA levels between these two values.

Main effect of general MPA level (i.e., DRES decreases with increasing general MPA level): We estimated the effect of general MPA level for each of the four combinations of session and time. While all four estimates were negative, the effect of general MPA level reached statistical significance only during the private performance and before the public performance. These results are detailed in Table 4 (see also Figure 1).

**Table 4:** Effect of general MPA level on DRES for each of the four combinations of session (private vs. public) and time (before performance vs. during performance).

	Coefficient	*SE*	*p*
Private session before	−0.024	0.014	0.085
Private session during	**−0.031**	**0.015**	**0.042**
Public session before	**−0.054**	**0.013**	**< 0.001**
Public session during	−0.018	0.014	0.21

Significant effects are highlighted in bold. The coefficients correspond to the slopes of the lines represented in Figure 1.

Main effect of session (i.e., DRES is lower during the public session than the private session): Before the performance, the session effect was significant for general MPA levels above 36 and was not significant for general MPA levels below 36. During the performance, the session effect was significant for general MPA levels below 59 and was not significant for general MPA levels above 59.

Main effect of time (i.e., DRES is lower during the performance than before the performance): During the private session, the time effect was significant for general MPA levels above 35 and was not significant for general MPA levels below 35. During the public session, the time effect was significant for general MPA level below 52 and was not significant for general MPA levels above 52.

### 3.2 Negative thoughts

Descriptive statistics for negative thoughts are reported in Table 2. Preliminary analyses of potential control variables revealed significant effects of depressive symptoms, age, preparation, and session × order (see Supplementary Table S4). These effects were thus added to the model with general MPA level, session, and their interaction. The final model is reported in Table 5. The main effects of MPA and session were significant, while their interaction was not significant. Negative thoughts increased with higher general MPA level and were higher after the public performance than the private performance.

**Table 5:** Fixed effects of the final model for negative thoughts.

	Coefficient	*SE*	*t*	*p*
MPA	**0.014**	**0.004**	**3.38**	**0.001**
Session	**0.176**	**0.058**	**3.04**	**0.003**
Depressive symptoms	0.017	0.006	2.86	0.005
Age	−0.036	0.014	−2.61	0.010
Preparation	−0.058	0.046	−1.25	0.21
Order	−0.056	0.088	−0.64	0.53
MPA × session	0.006	0.005	1.18	0.24
Session × order	0.260	0.174	1.47	0.14

MPA = general music performance anxiety level. For Session, the reference is the private session. For Order, the reference is private-public. Significant effects of interest are highlighted in bold.

### 3.3 Positive thoughts

Descriptive statistics for positive thoughts are reported in Table 2. Preliminary analyses of potential control variables revealed a significant effect of preparation (see Supplementary Table S5). This effect was thus included in the model with general MPA level, session, and their interaction. The final model is reported in Table 6. Only the effect of general MPA level was significant. Positive thoughts decreased with higher general MPA level.

**Table 6:** Fixed effects of the final model for positive thoughts.

	Coefficient	*SE*	*t*	*p*
MPA	**−0.018**	**0.007**	**−2.61**	**0.010**
Session	0.014	0.071	0.20	0.84
Preparation	0.075	0.038	1.98	0.050
MPA x session	0.003	0.006	0.53	0.67

MPA = general music performance anxiety level. For Session, the reference is the private session. Significant effects of interest are highlighted in bold.

### 3.4 Link between DRES and post-performance thoughts

The final PSRs were 1.001 for the model testing the effect of pre-performance DRES and 1.002 for the model testing the effect of during-performance DRES. These values suggest that the estimation of the two MCMC chains converged successfully (Hamaker et al., 2018). Inspection of the posterior parameter trace plots and autocorrelation plots showed no irregularities.

The results of the two-level path analyses are reported in Table 7. At the within-person level, the results indicated nonsignificant effects of pre-performance DRES on both negative thoughts and positive thoughts. In contrast, during-performance DRES significantly predicted fewer negative thoughts and more positive thoughts. At the between-person level, both pre-performance and during-performance DRES predicted fewer negative thoughts and more positive thoughts.

**Table 7:** Unstandardized and standardized effects (estimates and 95% HPD-CIs) of pre-performance DRES and during-performance DRES on negative and positive thoughts at the within-person and between-person levels.

	Pre-performance DRES		During-performance DRES	
	Unstandardized	Standardized	Unstandardized	Standardized
Within level				
DRES → negative thoughts	−0.05 [−0.13, 0.03]	−0.11 [−0.24, 0.04]	**−0.19** **[−0.27, −0.12]**	**−0.44** **[−0.56, −0.31]**
DRES → positive thoughts	−0.01 [−0.11, 0.09]	−0.02 [−0.16, 0.13]	**0.20** **[0.10, 0.31]**	**0.34** **[0.21, 0.48]**
Between level				
DRES → negative thoughts	**−0.18** **[−0.26, −0.09]**	**−0.49** **[−0.68, −0.28]**	**−0.13** **[−0.20, −0.07]**	**−0.45** **[−0.64, −0.24]**
DRES → positive thoughts	**0.27** **[0.14, 0.41]**	**0.44** **[0.24, 0.63]**	**0.27** **[0.16, 0.37]**	**0.50** **[0.31, 0.68]**

Effects whose 95% CI does not contain zero are significant and highlighted in bold.

## 4 Discussion

### 4.1 Effects of general MPA level, audience, and time on DRES

We examined how DRES—a measure that captures challenge versus threat states, with lower values reflecting a greater sense of threat—varied as a function of general MPA level, audience presence, and time (before vs. during performance). We hypothesized that DRES would be lower in the public session compared to the private one, decrease as general MPA levels increase, particularly in the public session, and be lower during performances than before. While these expectations were largely confirmed, the analyses revealed a more complex DRES pattern, with the three factors interacting with each other in shaping participants’ demand-resource evaluations.

DRES decreased as general MPA levels increased, indicating that higher general MPA levels were associated with lower DRES, a finding that aligns with research in the social anxiety literature (Gramer et al., 2012; Jamieson et al., 2013) and MPA literature (Craske and Craig, 1984). However, a novel contribution of this study is the finding that the strength of this association depended on when DRES was assessed. As shown in Table 4, the difference in DRES between participants with lower and higher general MPA levels was largest before the public performance. This pattern aligns with the conceptualization of anxiety as “… a future-oriented mood state associated with preparation for possible, upcoming negative events” (Craske et al., 2009, p. 1067). Accordingly, it is plausible that MPA manifests most strongly in the anticipation of performing in front of an audience. Interestingly, this finding contrasts with Gramer et al. (2012), who found that differences in DRES between participants with lower and higher social anxiety were larger during-speech than pre-speech. This discrepancy may reflect differences in task context across studies, underscoring the importance of further investigating the dynamic nature of DRES in performance settings.

Regarding the audience effect on DRES, we found, as predicted and consistent with finding from the social anxiety literature (Jamieson et al., 2013), a main session effect, indicating that DRES was lower in the public session than in the private session. This effect is likely driven by perceived social evaluation, which is particularly intense in performance settings (Rohleder et al., 2007; Kemeny, 2009). Importantly, the strength of this session effect varied across the continuum of general MPA level and differed before and during performance. Before the performance, the session effect was stronger at higher general MPA levels, as indicated by a significant general MPA level x session interaction. As shown in Figure 1, at lower general MPA levels, DRES was relatively high and similar in both private and public sessions (the session effect was not significant for general MPA levels below 36). In contrast, at higher general MPA levels, there was a substantial drop in DRES from the private to the public session. In other words, the shift toward threat appraisal in anticipation of audience evaluation increased with increasing general MPA level. During the performance, the pattern changed: at lower general MPA levels, the session effect was stronger, with a larger drop in DRES from the private to the public session. As general MPA level increased, this drop became progressively smaller, with the session effect becoming nonsignificant for general MPA levels above 59. However, the moderating effect of general MPA level during the performances was smaller than the one observed before the performances.

As predicted, participants reported lower DRES during the performance than before, consistent with findings from Aldao et al. (2014), Yeager et al. (2016), and Jacquart et al. (2020). However, this decline was nuanced by general MPA level and session. In the private session, the decline in DRES from pre-performance to during-performance was similar across general MPA levels, as indicated by a nonsignificant general MPA level x time interaction (and nonsignificant only for general MPA levels below 35). In contrast, in the public session, this interaction was significant, reflecting that at lower general MPA levels, there was a large drop in DRES, which became progressively smaller with increasing general MPA level, with the effect becoming nonsignificant for general MPA levels above 52.

### 4.2 Effects of general MPA level and audience on negative and positive thoughts

The second aim was to explore how participants’ general MPA level and audience presence influenced their negative and positive post-performance thoughts. We hypothesized that participants would report more negative thoughts and fewer positive thoughts after the public performance compared to the private performance. Additionally, we expected that as general MPA levels increase, participants would report more negative thoughts and fewer positive thoughts, especially after the public performance.

As hypothesized, negative thoughts were significantly higher following the public session compared to the private session. In our study, this pattern suggests that participants were more likely to perceive their performance negatively and engage in ruminative thoughts shaped by their belief that they had been evaluated unfavorably (Clark and Wells, 1995). In contrast, positive thoughts were not significantly different between the two performance sessions, which might reflect their lower sensitivity to contextual variations, such as audience presence, compared to negative thoughts.

Additionally, negative thoughts increased, while positive thoughts decreased with higher general MPA levels. This finding replicates Nielsen et al. (2018), who observed that higher general MPA levels among classical music students were significantly associated with more negative and fewer positive thoughts following a solo concert. The positive relationship between social anxiety and negative post-event thoughts has been consistently demonstrated across studies (Edgar et al., 2024). However, the relationship between social anxiety and positive post-event thoughts has yielded mixed results: some studies report a significant relationship (Kocovski et al., 2011; Gramer et al., 2012; Kane et al., 2023), while others do not (Edwards et al., 2003; Abbott and Rapee, 2004; Dannahy and Stopa, 2007). Further research is needed to reconcile these contrasting findings. Differences in methodology, including the instruments used to assess social anxiety, sample size, and procedural characteristics (e.g., whether post-event thoughts are assessed shortly after the social stressor or several days later), may contribute to the variability in results. Understanding these factors is crucial for clarifying the relationship between social anxiety and positive post-event thoughts.

Contrary to our expectations, session and general MPA level did not significantly interact in predicting post-performance thoughts. In other words, the heightened negative thoughts and reduced positive thoughts associated with higher general MPA levels appear to be primarily driven by individual differences in general MPA level, rather than the presence or absence of an audience. This result may be explained by the strong correlation between general MPA level and trait worry (*r* = 0.67), as highlighted by Nielsen et al. (2018). For high-anxious individuals, the generalized tendency to worry may exert a pervasive cognitive influence, dominating their post-event thought processes and minimizing the impact of situational variations like audience presence.

### 4.3 Absolute levels of DRES and post-performance thoughts

The results discussed in sections 4.1 and 4.2 illustrate how demand-resource evaluations and post-performance thoughts vary as a function of general MPA level, audience presence, and time point. Equally important is examining these results in absolute terms: How high or low were participants’ DRES and post-performance thoughts? Were participants predominantly in a threat or a challenge state? Did they experience more negative thoughts or positive thoughts overall?

Although further research is needed to refine the interpretation of DRES, it is generally accepted that a negative DRES reflects a threat state, while a positive DRES indicates a challenge state (Moore et al., 2018; Wood et al., 2018; Peek et al., 2023). As shown in Table 2, all four DRES means were positive, with three significantly exceeding zero. These findings suggest that, on average, participants were more often in a challenge state than a threat state, even during the public performance session.

Regarding post-performance processing, the mean scores for negative thoughts were lower than the mean scores for positive thoughts. Similar to the DRES results, these findings suggest a relatively positive outlook, as participants reported comparatively lower levels of negative thoughts and higher levels of positive thoughts overall.

### 4.4 DRES as a predictor of negative and positive post-performance thoughts

The third study’s aim was to examine how pre-performance and during-performance DRES predict negative and positive post-performance thoughts at both within-person and between-person levels. We hypothesized that higher DRES would predict fewer negative thoughts and more positive thoughts at both levels.

At the within-person level, pre-performance DRES did not significantly predict negative or positive thoughts, suggesting limited influence of initial challenge and treat appraisals on post-performance thoughts. In contrast, high during-performance DRES significantly predicted fewer negative thoughts and more positive thoughts.

At the between-person level, higher pre-performance and during-performance DRES were linked to fewer negative and more positive post-performance thoughts. This suggests that individuals with consistently high DRES adopt more suitable cognitive appraisals, which in turn result in more positive and less negative reflections after performance.

To the best of our knowledge, this is the first study to show that DRES as a self-report index of challenge and threat states predicts negative and positive post-task processing. These findings extend previous work that has shown that DRES predicts adaptive cardiovascular responses (Moore et al., 2014), as well as confidence and dominance during performance (Brimmell et al., 2018). DRES has also been shown to enhance attentional control (Vine et al., 2013) and promote more positive affect while reducing self-focused attention in high-pressure tasks (Wood et al., 2018). Collectively, these studies highlight the utility of DRES as a robust indicator of cognitive, emotional, and physiological responses across various contexts.

Our findings align with the integrated framework, connecting the biopsychosocial model of challenge and threat to the extended perseverative cognition hypothesis. This integration underscores the dynamic interplay between demand and resource appraisals and subsequent cognitive processes, such as post-performance thoughts. This framework is also valuable in differentiating within-subject and between-subject levels. Future studies could build on this framework to uncover additional mechanisms underlying post-event thoughts and their variability across contexts.

### 4.5 Strengths, limitations, and outlook

The present study included a large sample of classical music students and employed an experimental design featuring a familiarization session and two counterbalanced performance sessions. The multi-item questionnaires demonstrated good to excellent reliability, and advanced analytical methods and the consideration of several potential control variables ensured robust testing of the hypotheses.

Despite these strengths, certain limitations warrant consideration. First, the findings are influenced by the characteristics of the instruments used. Consistent with prior studies (Nielsen et al., 2018; Guyon et al., 2020a), we assessed general MPA level using the STAI-S. However, other MPA-specific questionnaires, such as the Kenny Music Performance Anxiety Inventory (Kenny et al., 2014) and the Performance Anxiety Questionnaire (Cox and Kenardy, 1993), are available. Although these measures are significantly correlated with one another (Kenny et al., 2004; Antonini Philippe et al., 2022), they are grounded in different theoretical models and may capture distinct aspects of MPA. Future research could explore whether using MPA-specific instruments provides additional insights into the relationship between MPA, demand and resource evaluations, and post-performance thoughts. Similarly, while the DRES has demonstrated strong conceptual and predictive validity (e.g., Moore et al., 2013; Hase et al., 2019) and its brevity makes it particularly appealing for studies with time constraints, the assessment of challenge and threat appraisals remains a topic of ongoing debate and research, with several alternative measures (Jacquart et al., 2020; Meijen et al., 2020; Grylls et al., 2021; Peters et al., 2024). Studies are needed to compare the DRES with other measures to evaluate their relative strengths and applicability. Additionally, the Post-Music Performance Thoughts Questionnaire, like the Thoughts Questionnaire from which it was derived (Edwards et al., 2003), assesses the presence of specific post-performance thoughts but does not examine characteristics inherent to post-event processing such as intrusiveness, repetitiveness, and uncontrollability (Rachman et al., 2000; Ehring et al., 2011; Flynn and Yoon, 2025). Developing a music performance-specific questionnaire that incorporates these features could provide a more comprehensive tool for examining the full scope of post-performance processing in musicians.

Second, this study examined intra-individual differences in the relationship between DRES and post-performance thoughts but relied on only two observations - private and public. While insightful, this design does not capture potential intra-variability in DRES and post-performance thoughts over time or across contexts. Future research should consider using ecologic momentary assessment to collect demand-resource evaluations and post-performance thoughts across a larger number of performance situations per musician. This approach would allow for more precise estimation of within-person relationships, the identification of temporal patterns, and the characterization of distinct profiles.

Third, while our study focused on quantitative measures, qualitative data—such as interviews or think-aloud protocols—could have provided deeper insight into the nature of participants’ experiences. Future research could integrate such methods to complement self-report questionnaires.

Fourth, this study was conducted with classical music students, a population for whom MPA represents a significant concern (Studer et al., 2011). Extending this line of research to other musician populations, as well as performers in disciplines such as dance and theatre, could help determine whether the observed patterns generalize across different artistic domains, performance contexts, and levels of expertise.

Finally, a promising avenue for future research lies in drawing conceptual and empirical connections between the biopsychosocial model of challenge and threat and flow theory (Csikszentmihalyi, 1997; Kirchner et al., 2008; Fullagar et al., 2013). Both frameworks emphasize the central role of the balance between situational demands and personal resources or skills in fostering optimal states of engagement. Indeed, challenge states in the biopsychosocial model of challenge and threat—marked by the perception of sufficient resources to meet task demands—conceptually align with the “challenge-skill balance” identified as a prerequisite for flow (Csikszentmihalyi, 1997). While a few scholars have explored these parallels (Mendes et al., 2003; Tozman and Peifer, 2016), the integration has remained largely theoretical. Future research could benefit from concurrently applying measures from both models—such as the DRES and the Flow State Scale (Jackson and Eklund, 2002)—to examine their interrelations and how they contribute to predicting outcomes such as post-performance processing and performance quality.

## 5 Conclusion and implications

This study demonstrated that as general MPA levels increased, participants reported lower DRES. Audience presence reduced DRES overall, with the effect varying by general MPA level and time point. Before the performance, higher general MPA levels were associated with a greater drop in DRES from private to public sessions, whereas lower general MPA levels were linked to a smaller effect. During the performance, the pattern shifted, with lower general MPA levels showing a larger decline in DRES across sessions compared to higher general MPA levels. DRES also declined from pre-performance to during-performance, with differences influenced by session type and general MPA level. In the private session, the decline was relatively uniform, whereas in the public one, lower general MPA levels were associated with a pronounced decrease, which diminished as general MPA levels increased. These findings highlight the dynamic effects of MPA, audience presence, and time on demand-resource evaluations.

Negative thoughts were higher after the public session compared to the private session, and higher general MPA levels were associated with more negative and fewer positive thoughts. Session type and general MPA levels did not interact significantly.

Additionally, the study explored for the first time how pre- and during-performance DRES influences post-event thoughts at both the within-person and between-person levels. Lower during-performance DRES predicted more negative and fewer positive post-performance thoughts both within and between individuals, whereas pre-performance DRES had a significant effect only at the between-subject level. This underscores the critical relationship between DRES and perseverative cognitions.

These findings have important implications for future research on music performance and MPA. Despite the growing interest in psychological factors affecting musicians, demand-resource evaluations and post-performance thoughts remain understudied. Future studies should integrate these measures more systematically to better understand how musicians appraise performance situations and how these appraisals shape their post-performance processing. Expanding research in this direction could provide valuable insights into the cognitive and emotional mechanisms underlying music performance anxiety and inform strategies to support musicians’ well-being.

These findings also have practical implications for interventions aimed at optimizing performance experiences. One promising approach is stress arousal reappraisal, which encourages individuals to reinterpret stress arousal as a functional resource rather than a sign of impending failure (Jamieson et al., 2018; Bosshard and Gomez, 2024). This method has been shown to be beneficial not only for anxious individuals but also for those with lower anxiety, suggesting its broad applicability in performance settings (Jamieson et al., 2013; Moore et al., 2015; Sharpe et al., 2024). Given that lower DRES during performance was strongly linked to more negative and fewer positive post-performance thoughts, interventions that help musicians perceive greater resources could have a lasting impact on their post-performance evaluations and overall well-being.

Building on this, future research could also explore how these insights might be translated into practical tools for performers. For instance, providing musicians with visual feedback on how their demand and resource evaluations shift across different performances could help them identify potentially maladaptive appraisal patterns. This feedback might then be integrated into interventions to promote more adaptive coping strategies and enhance performance outcomes.

## Data Availability

The raw data supporting the conclusions of this article will be made available by the authors, without undue reservation.
