# Supplementary material for: Demand-resource evaluations and post-performance thoughts in classical music students: how they are linked and influenced by music performance anxiety, audience, and time
Source: Front Psychol. 2025 May 12;16:1579759. doi: 10.3389/fpsyg.2025.1579759 (PMC12104281; doi:10.3389/fpsyg.2025.1579759)
Supplement: Supplementary file 1 [file Data_Sheet_1.pdf]

Supplementary Material

Demand-Resource Evaluations and Post-performance Thoughts in Classical Music Students: How They Are Linked and Influenced by Music Performance Anxiety, Audience, and Time

Ludovic Rey, Amélie J. A. A. Guyon, Horst Hildebrandt, Angelika Güsewell, Antje Horsch, Urs M. Nater, Jeremy P. Jamieson and Patrick Gomez

Table S1. Glossary of key psychological concepts.

Term / Concept	Definition / Description	Key References
**Music Performance Anxiety (MPA)**	A persistent and significant experience of anxious apprehension related to musical performance, often more intense in evaluative or high-stakes settings.	Kenny, 2010; Studer et al., 2011
**Biopsychosocial Model of Challenge and Threat**	A framework describing how individuals evaluate performance situations as either a **challenge** (resources ≥ demands) or a **threat** (demands > resources).	Blascovich & Mendes, 2000; Seery, 2013
**Demand-Resource Evaluation Score (DRES)**	A continuous measure representing the subjective difference between perceived personal resources and perceived situational demands. Higher scores indicate greater challenge.	Moore et al., 2014; Peek et al., 2023
**Transactional Model of Stress and Coping**	A theory positing that stress results from how individuals appraise and cope with external demands in relation to their perceived coping resources.	Lazarus & Folkman, 1984
**Physiological Toughness**	A concept describing the body's adaptive responses (e.g., cardiovascular) to stress, influenced by appraisals and experience.	Dienstbier, 1989
**Perseverative Cognition**	Repetitive or sustained thoughts about past or future stressors, which can be negatively or positively valenced, and impact mental and physical health.	Brosschot et al., 2010; Ottaviani et al., 2016
**Post-Performance Thoughts**	Self-referential reflections after performing, which can be either negative (e.g., "I made mistakes") or positive (e.g., "That went well").	Nielsen et al., 2018; Haccoun et al., 2020
**Post-Event Processing**	A cognitive process typically involving negative review of past social or performance events, contributing to anxiety maintenance.	Clark & Wells, 1995; Flynn & Yoon, 2025

Table S2. Means and standard deviations of the demand and resource evaluations during the private and public sessions.

	Private session		Public session	
	Before	During	Before	During
Demand evaluation	3.55 (1.30)	3.49 (1.38)	4.00 (1.12)	4.12 (1.08)
Resource evaluation	4.83 (0.85)	4.22 (1.15)	4.55 (0.95)	4.23 (1.09)

Preliminary analysis of the effects of the potential control variables on DRES

Table S3. Estimated effects (main effects, 2-way interactions, and 3-way interactions with session and time) of the potential control variables on ***DRES***.

	Coefficient	*SE*
Gender	**0.500**	**0.253**
Age	0.013	0.040
Depressive symptoms	-0.017	0.016
Order	0.341	0.253
Time of day	0.207	0.254
Time difference	0.003	0.020
Preparation	-0.018	0.058
Gender x session	0.160	0.208
Age x session	-0.015	0.032
Depressive symptoms x session	-0.021	0.013
Order x session	-0.009	0.206
Time of day x session	0.076	0.206
Time difference x session	0.012	0.016
Gender x time	-0.124	0.216
Age x time	0.030	0.034
Depressive symptoms x time	0.015	0.014
Order x time	-0.289	0.214
Time of day x time	0.280	0.214
Time difference x time	-0.007	0.017
Gender x session x time	-0.005	0.412
Age x session x time	0.052	0.064
Depressive symptoms x session x time	0.027	0.026
Order x session x time	0.022	0.407
Time of day x session x time	-0.392	0.406
Time difference x session x time	0.007	0.032

Note. Each effect was tested individually. Significant effects ($p < 0.05$) are highlighted in bold. Note that *preparation* was tested only as a main effect as it is a within-person variable. Reference categories for the categorical variables are as follows: *gender*: female; *order*: private-public; *time of day*: early afternoon; *session*: private; *time*: before the performance. Units for the continuous variables are as follows: *age*: 1 year; *depressive symptoms*: 1 scale point; *time difference*: 10 days; *preparation*: 1 h. *SE* = standard error.

Preliminary analysis of the effects of the potential control variables on negative thoughts

Table S4. Estimated effects (main effects and 2-way interactions with session) of the potential control variables on ***negative thoughts***.

	Coefficient	*SE*
Gender	-0.160	0.097
Age	**-0.032**	**0.015**
Depressive symptoms	**0.021**	**0.006**
Order	-0.023	0.097
Time of day	-0.109	0.097
Time difference	-0.001	0.001
Preparation	**-0.107**	**0.032**
Gender x session	0.100	0.122
Age x session	0.012	0.019
Depressive symptoms x session	0.001	0.008
Order x session	**0.421**	**0.115**
Time of day x session	0.048	0.121
Time difference x session	-0.001	0.001

Note. Each effect was tested individually. Significant effects ($p < 0.05$) are highlighted in bold. Note that *preparation* was tested only as a main effect as it is a within-person variable. Reference categories for the categorical variables are as follows: *gender*: female; *order*: private-public; *time of day*: early afternoon; *session*: private. Units for the continuous variables are as follows: *age*: 1 year; *depressive symptoms*: 1 scale point; *time difference*: 10 days; *preparation*: 1 h. *SE* = standard error.

Preliminary analysis of the effects of the potential control variables on positive thoughts

Table S5. Estimated effects (main effects and 2-way interactions with session) of the potential control variables on ***positive thoughts***.

	Coefficient	*SE*
Gender	0.159	0.154
Age	0.026	0.024
Depressive symptoms	-0.015	0.010
Order	-0.093	0.153
Time of day	0.011	0.153
Time difference	0.002	0.001
Preparation	**0.075**	**0.037**
Gender x session	-0.127	0.143
Age x session	0.001	0.022
Depressive symptoms x session	0.007	0.009
Order x session	-0.197	0.141
Time of day x session	-0.076	0.142
Time difference x session	0.001	0.001

Note. Each effect was tested individually. Significant effects ($p < 0.05$) are highlighted in bold. Note that *preparation* was tested only as a main effect as it is a within-person variable. Reference categories for the categorical variables are as follows: *gender*: female; *order*: private-public; *time of day*: early afternoon; *session*: private. Units for the continuous variables are as follows: *age*: 1 year; *depressive symptoms*: 1 scale point; *time difference*: 10 days; *preparation*: 1 h. *SE* = standard error.

Analysis of the secondary variables demand evaluation and resource evaluation

The analysis of demand evaluation and resource evaluation was carried out like the analysis of DRES described in the paper in terms of predictors of interest and potential control variables. Regarding the random effect structure, the model for demand evaluation included a random intercept for participants and a random coefficient for session with unstructured covariance, and the residual variance structure was homogeneous (i.e., one common variance). The model for resource evaluation included a random intercept, and the residual variance structure was heterogeneous (distinct variance for each time).

Results for demand evaluation

Preliminary analysis of the effects of the potential control variables on demand evaluation

Table S6. Estimated effects (main effects, 2-way interactions, and 3-way interactions with session and time) of the potential control variables on ***demand evaluation*.**

	Coefficient	*SE*
Gender	-0.132	0.180
Age	0.035	0.028
Depressive symptoms	0.003	0.012
Order	**-0.519**	**0.173**
Time of day	-0.156	0.178
Time difference	-0.002	0.014
Preparation	**0.099**	**0.048**
Gender x session	-0.164	0.166
Age x session	-0.009	0.026
Depressive symptoms x session	0.017	0.011
Order x session	**-0.378**	**0.162**
Time of day x session	-0.188	0.164
Time difference x session	-0.005	0.013
Gender x time	-0.070	0.132
Age x time	0.011	0.020
Depressive symptoms x time	0.007	0.008
Order x time	0.112	0.124
Time of day x time	-0.213	0.123
Time difference x time	0.007	0.010
Gender x session x time	-0.353	0.249
Age x session x time	-0.075	0.039
Depressive symptoms x session x time	-0.019	0.016
Order x session x time	0.178	0.247
Time of day x session x time	0.472	0.244
Time difference x session x time	-0.001	0.019

Note. Each effect was tested individually. Significant effects ($p < 0.05$) are highlighted in bold. Note that *preparation* was tested only as a main effect as it is a within-person variable. Reference categories for the categorical variables are as follows: *gender*: female; *order*: private-public; *time of day*: early afternoon; *session*: private; *time*: before the performance. Units for the continuous

variables are as follows: *age*: 1 year; *depressive symptoms*: 1 scale point; *time difference*: 10 days; *preparation*: 1 h. *SE* = standard error.

Main analysis

Preliminary analyses of potential control variables revealed significant effects of order, preparation, and Session x order interaction (see Table S6). These effects were thus added to the main model alongside general MPA level, session, time, and their interactions. The final model is reported in Table S7.

Table S7. Fixed effects of the final model for demand evaluation.

	Coefficient	*SE*	*t*	*p*
MPA	0.012	0.008	1.37	0.17
Session	**0.558**	**0.082**	**6.77**	**< 0.001**
Time	0.033	0.062	0.53	0.59
Order	-0.380	0.185	-2.06	0.042
Preparation	-0.013	0.062	-0.21	0.83
MPA x session	0.006	0.008	0.80	0.43
MPA x time	-0.006	0.006	-1.11	0.27
Session x time	0.182	0.124	1.47	0.14
Session x order	-0.418	0.239	-1.75	0.08
MPA x session x time	-0.018	0.011	-1.62	0.11

Note. MPA = general music performance anxiety level. For Session, the reference is the private session. For Time, the reference is before the performance. For Order, the reference is private-public. Significant effects of interest are highlighted in bold.

Results for resource evaluation

Preliminary analysis of the effects of the potential control variables on resource evaluation

Table S8. Estimated effects (main effects, 2-way interactions, and 3-way interactions with session and time) of the potential control variables on ***resource evaluation***.

	Coefficient	*SE*
Gender	**0.405**	**0.136**
Age	**0.050**	**0.022**
Depressive symptoms	**-0.019**	**0.009**
Order	-0.028	0.140
Time of day	0.105	0.140
Time difference	0.001	0.011
Preparation	**0.075**	**0.036**
Gender x session	0.074	0.132
Age x session	-0.014	0.021
Depressive symptoms x session	-0.002	0.008
Order x session	**-0.429**	**0.128**
Time of day x session	-0.173	0.131
Time difference x session	0.007	0.010
Gender x time	-0.065	0.138
Age x time	0.037	0.021
Depressive symptoms x time	**0.020**	**0.009**
Order x time	-0.178	0.136
Time of day x time	0.093	0.136
Time difference x time	-0.001	0.011
Gender x session x time	-0.358	0.272
Age x session x time	-0.024	0.042
Depressive symptoms x session x time	0.008	0.017
Order x session x time	0.200	0.266
Time of day x session x time	0.080	0.269
Time difference x session x time	0.007	0.021

Note. Each effect was tested individually. Significant effects ($p < 0.05$) are highlighted in bold. Note that *preparation* was tested only as a main effect as it is a within-person variable. Reference categories for the categorical variables are as follows: *gender*: female; *order*: private-public; *time of day*: early afternoon; *session*: private; *time*: before the performance. Units for the continuous variables are as follows: *age*: 1 year; *depressive symptoms*: 1 scale point; *time difference*: 10 days; *preparation*: 1 h. *SE* = standard error.

Main analysis

Preliminary analyses of potential control variables revealed significant effects of gender, age, depressive symptoms, order, preparation, session x order, and time x depressive symptoms (see Table S8). These effects were thus added to the main model, alongside general MPA level, session, time, and their interaction. The final model is reported in Table S9.

Table S9. Fixed effect of the final model for resource evaluation.

	Coefficient	*SE*	*t*	*p*
MPA	**-0.021**	**0.006**	**-3.25**	**< 0.001**
Session	-0.119	0.067	-1.77	0.08
Time	**-0.463**	**0.067**	**-6.94**	**< 0.001**
Gender	0.202	0.137	1.47	0.14
Age	0.047	0.021	2.30	0.024
Depressive symptoms	-0.006	0.009	-0.69	0.50
Order	0.019	0.129	0.15	0.88
Preparation	-0.016	0.048	-0.34	0.74
Session x order	-0.468	0.181	-2.59	0.010
Time x depressive symptoms	0.018	0.010	2.03	0.043
MPA x session	-0.002	0.006	-0.39	0.70
MPA x time	0.005	0.006	0.79	0.43
Session x time	**0.281**	**0.133**	**2.11**	**0.035**
MPA x session x time	**0.025**	**0.012**	**2.10**	**0.036**

Note. MPA = general music performance anxiety level. For Session, the reference is the private session. For Time, the reference is before the performance. For Order, the reference is private-public. Significant effects of interest are highlighted in bold.
